# MSC Therapy Attenuates Obliterative Bronchiolitis after Murine Bone Marrow Transplant

**DOI:** 10.1371/journal.pone.0109034

**Published:** 2014-10-01

**Authors:** Kashif Raza, Trevor Larsen, Nath Samaratunga, Andrew P. Price, Carolyn Meyer, Amy Matson, Michael J. Ehrhardt, Samuel Fogas, Jakub Tolar, Marshall I. Hertz, Angela Panoskaltsis-Mortari

**Affiliations:** 1 Pulmonary, Allergy, Critical Care and Sleep Medicine, University of Minnesota, Minneapolis, Minnesota, United States of America; 2 Breck High School, Edina, Minnesota, United States of America; 3 Pediatric Blood and Bone Marrow Transplant Program, University of Minnesota Cancer Center, Minneapolis, Minnesota, United States of America; Cedars-Sinai Medical Center, United States of America

## Abstract

**Rationale:**

Obliterative bronchiolitis (OB) is a significant cause of morbidity and mortality after lung transplant and hematopoietic cell transplant. Mesenchymal stromal cells (MSCs) have been shown to possess immunomodulatory properties in chronic inflammatory disease.

**Objective:**

Administration of MSCs was evaluated for the ability to ameliorate OB in mice using our established allogeneic bone marrow transplant (BMT) model.

**Methods:**

Mice were lethally conditioned and received allogeneic bone marrow without (BM) or with spleen cells (BMS), as a source of OB-causing T-cells. Cell therapy was started at 2 weeks post-transplant, or delayed to 4 weeks when mice developed airway injury, defined as increased airway resistance measured by pulmonary function test (PFT). BM-derived MSC or control cells [mouse pulmonary vein endothelial cells (PVECs) or lung fibroblasts (LFs)] were administered. Route of administration [intratracheally (IT) and IV] and frequency (every 1, 2 or 3 weeks) were compared. Mice were evaluated at 3 months post-BMT.

**Measurements and Main Results:**

No ectopic tissue formation was identified in any mice. When compared to BMS mice receiving control cells or no cells, those receiving MSCs showed improved resistance, compliance and inspiratory capacity. Interim PFT analysis showed no difference in route of administration. Improvements in PFTs were found regardless of dose frequency; but once per week worked best even when administration began late. Mice given MSC also had decreased peribronchiolar inflammation, lower levels of hydroxyproline (collagen) and higher frequencies of macrophages staining for the alternatively activated macrophage (AAM) marker CD206.

**Conclusions:**

These results warrant study of MSCs as a potential management option for OB in lung transplant and BMT recipients.

## Introduction

Obliterative bronchiolitis (OB) is a significant problem in lung transplant and BMT recipients. OB is directly or indirectly responsible for almost 40% of lung transplant related deaths [1]. This is mainly due to chronic allograft dysfunction, manifesting as OB, characterized histologically by inflammation and fibrosis of small airways. In BMT recipients, the incidence of OB has been reported to be as high as 29% with increased risk of mortality and is associated with chronic graft-versus-host disease (GVHD) [2], [3]. After transplant, the host immune system is activated by exposure to allogeneic tissue antigens, resulting in an inflammatory cascade with alloimmune and non-alloimmune dependent factors contributing to the response. The cumulative end result of this cascade is OB [4]. Current management strategies involving immunosuppressive medications have not been very successful.

Lack of suitable animal models has limited efforts to understand and develop therapeutic strategies for OB. We have previously reported a new murine BMT model, in which chronic GVHD leads to OB similar to the chronic rejection seen in lung transplantation [5].

MSCs provide a promising management option for this population. They have immunomodulatory properties, among which is their ability to suppress T-lymphocyte activation and proliferation, key events in allograft rejection [6]. MSCs have been shown to inhibit maturation of dendritic cells and promote secretion of anti-inflammatory cytokines, resulting in generation of Tregs(reviewed in [7]). Tregs can suppress effector FoxP3^negative^ cells and antigen presenting cells (APCs) thereby inhibiting inflammatory responses. MSCs and MSC-induced Tregs are capable of generating alternatively activated macrophages (AAMs), which are immunosuppressive and inhibit the proliferation of activated CD4^+^ T cells [8].

MSCs have been used successfully to prolong allograft survival in other animal models of organ transplantation [9], [10], [11]. Donor human lungs (rejected for transplant) infused with MSCs have improved alveolar fluid clearance compared to the current state of the art technique [12]. In the context of BMT, MSCs have shown efficacy in ameliorating graft-versus-host-disease (GVHD) [13], [14], [15] and have been approved for steroid-refractory acute GVHD. They have been used safely as a co-infusion in patients undergoing unrelated allogeneic bone marrow transplant [16]. MSCs have not been previously evaluated as a cell therapy for OB post-BMT although they have been studied many times in other lung injury models where they are given as either a pretreatment or concomitantly with injury induction (reviewed in [17], [18], [19]. A number of clinical trials in a variety of lung diseases are underway using these cells to further establish their safety and efficacy [20]. In the present study, we tested the hypothesis that exogenous MSCs will reduce the occurrence and severity of OB in our murine model. We found that administration of MSCs attenuated injury and airway lumen obliteration and led to improvement of lung function even if given after lung function had declined.

## Methods

### Ethics Statement

All experiments were approved by the University of Minnesota Institutional Animal Care and Use Committee (assurance #A3456-01, IACUC # 0906A67041).

### Bone marrow transplantation

Our BMT protocol has been described [5]. Recipient female B10.BR mice [lethally conditioned with cyclophosphamide 120 mg/kg/d on days -3,-2 (Bristol Myers Squibb, Seattle, WA) and 7.5 Gy irradiation on day -1] were given male C57BL/6J (B6) donor BM (15×10^6^, T-cell depleted) without or with 2×10^6^ B6 spleen cells via caudal vein. All experiments were approved by the University of Minnesota Institutional Animal Care and Use Committee (assurance #A3456-01, IACUC # 0906A67041). The results of 5 transplant experiments were used for this study, with each experiment consisting of at least 5 mice per group.

### Isolation of MSCs

MSCs were isolated from male B6 GFP transgenic mice as described [21]. After 4 weeks of initial culture, adherent cells were plated at 50 cells/cm^2^ in Iscove’s modified Dulbecco medium (Invitrogen), 9% FCS, 9% horse serum, pen-strep, and 2 µM l-glutamine. Cells were expanded at low density (50–100 cells/cm^2^) with medium replaced every 3–4 days. Consistent with the reported results of the Prockop lab [21], the MSCs demonstrated high expression of CD106 (86%), Sca-1 & CD44 (both 98%), low/very low expression of CD45 (3–4%), CD31 (<2%), CD117 (2%), CD90 (<2%) and MHC I (13%) (Figure S1). They were negative for MHC II. MSCs displayed osteocyte, chondrocyte and adipocyte trilineage differentiation as we have previously shown [22].

### Control cells

Since many cell types can produce beneficial mediators, control cells were used to demonstrate the specificity of the cell therapy effect to MSCs.

PVECs were isolated in the lab of Dr. Robert Hebbel (UMN). Briefly, pulmonary vein from B6 mice was cut into pieces and treated with collagenase II. PVECs were grown using the method developed for microvascular endothelial cells [23]. The resulting cells were identified as endothelial by positive staining for CD31, von Willebrand factor, VE-cadherin, VCAM-1, and flk-1; and negative staining for vimentin and smooth-muscle actin.

Mouse lung fibroblasts (LFs) were isolated as described [24] from B6 mice. Fibroblasts were maintained in DMEM, 10% FBS, L-glutamine and pen-strep until time of administration.

### Administration of experimental and control cells

Cohorts of mice receiving BMS received either 10^4^ passage 2–5 MSCs or control cells in a 50 µL aliquot of culture medium intra-tracheally (IT, under anesthesia with intubation under direct visualization) or IV starting at either 2 weeks or IV starting at 4 weeks post-BMT and continued up to 14 weeks post-BMT (given every 1, 2 or 3 weeks). BMS mice not receiving cell therapy were given non-conditioned medium alone.

### Pulmonary Function Tests

PFTs were assessed by whole body plethysmography using the Flexivent system (Scireq, Montreal, PQ, Canada) as described previously [5]. The maximum pressure was set to 30 cmH_2_O. The positive end-expiratory pressure remained constant at 2.5 cm H_2_O.

### Histopathology and OH-proline Quantification

Tissue preparation was done as described [5]. Cryosections (6 µm) were stained by H&E for pathological analysis. Lung pathology was assessed using a semi-quantitative (0–4 grade) scoring system [5]. Four sections from each lung were evaluated. OH-proline levels were determined by oxidation of 4-OH-L-proline to pyrrole and reaction with *p*-dimethylaminobenzaldehyde (absorbance read at 560 nm).

### Immunofluorescence

Immunofluorescence staining was used to detect AAMs and Tregs on serial sections as described [25]. Tregs were detected using biotinylated anti-FoxP3 (FJK16s; eBioscience) with streptavidin-cyanin 3 (Jackson ImmunoResearch) and FITC-anti-CD4. Macrophages were stained with CD11c (BDPharmingen) for conventional M1 macrophages and polyclonal rabbit IgG anti-CD206 (Santa Cruz) followed by cyanin 3-labeled anti-rabbit antibody (Jackson) to identify AAMs (M2 macrophages). Images were acquired on an Olympus FluoView 500 BX51 confocal microscope and FluoView software version 4.3.

### Flow Cytometry

Antibodies with specificity for CD4, FoxP3, CD 206 and F4/80 were obtained from eBioscience. For cell number quantification, PE, PerCP and APC labeled counting beads (Invitrogen) were used. Explanted lungs were digested in collagenase containing PBS for 60 minutes at 37C. Single cell suspension was obtained by passing the cells through 40 um cell strainer (BD). For intracellular cytokine staining, cells were stained for CD4, fixed, and permeabilized using the Fix&Perm kit (BD) and stained with antibody to FoxP3. Cells were analyzed on a 4-color FACS Calibur instrument (BD) with FlowJo version 8.8.

### qRT-PCR

Total RNA was extracted from lungs on day 90 post-BMT using Trizol (Life Technologies, Grand Island, NY), purified using the PureLink RNA MiniKit (Life Technologies), and cDNA generated by reverse transcription with the Superscript III kit (Invitrogen) following manufacturer’s instructions. Quantitative real-time PCR was performed on an ABI 7500 Real Time PCR System using TaqMan Gene Expression Master Mix (Applied Biosystems). Mouse probes for GAPDH (Mm99999915_g1), IL-10 (Mm00439615_g1) and CD206 (Mm01329362_m1) were purchased from Life Technologies (Grand Island, NY).

### Cytokine Analysis

Cell culture supernatants (48-hours) were analyzed in duplicate by ELISA using kits: PGE2 (multi-species), rat IL-1RA, rat IL-10 (R&D Systems, Minneapolis, MN); FGF7/KGF (BlueGene, Shanghai); MPO (RayBioTech, Norcross, GA); TSG6 (MyBiosource, San Diego, CA). Other cytokines were evaluated on a Luminex (Austin, TX) using rat-specific bead sets: FasL, G-CSF, GM-CSF, IFNγ, IL-4, IL-12p40&p70, LIX, RANTES (Millipore, Billerica, MA); PAI-1, CXCL1, TNFα, IL1α, IL-2, IL-4, IL-6 (R&D Systems).

### Statistical Analysis

Data were analyzed by analysis of variance or *t* test with significance at *P*≤0.05. Numerical data are shown as the mean ± standard error.

## Results

### Determination of time points for cell therapy intervention for OB

**Figure 1:**
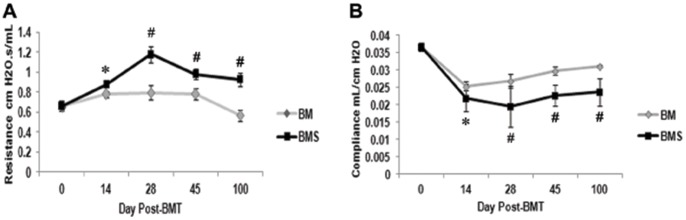
Kinetics of pulmonary function parameters post-BMT show that OB manifestations begin as early as 2 weeks. Lethally conditioned B10.BR mice were given B6 BM with (▪BMS) or without (◊BM) spleen cells. PFTs were done on the days indicated sequentially on the same mice. **A**. Resistance; **B**. Compliance. *p<0.05 vs day 0 for BMS (OB) group. #p<0.05 BMS vs BM group. N = 5–40/group/time point pooled from 4 experiments.

To understand the kinetics of OB development, and to define time points for intervention in each experiment, PFTs were done in individual mice sequentially at defined time points post-BMT. (**Figure 1** **)** In the BMS mice (OB group), lung resistance and compliance deteriorated from 2 weeks post-BMT and diverged from the BM group at 4 weeks post-BMT. All mice, including those receiving BM only, have some reduction in lung function early after BMT; i.e. BMS mice were not statistically different from BM mice at 2 weeks. This resolves in BM mice as they recover and are hematopoietically rescued. Thus, an intervention would be more meaningful if it could be done when the beginning of OB manifestations can be clearly identified. In subsequent experiments, we considered these two times as points of intervention, with the 2 week point as “early” and the 4 week time point as “late” cell therapy. The initial decline in lung function by PFTs was confirmed for every mouse prior to cell therapy.

### Early administration of MSCs resolves OB after BMT

**Figure 2:**
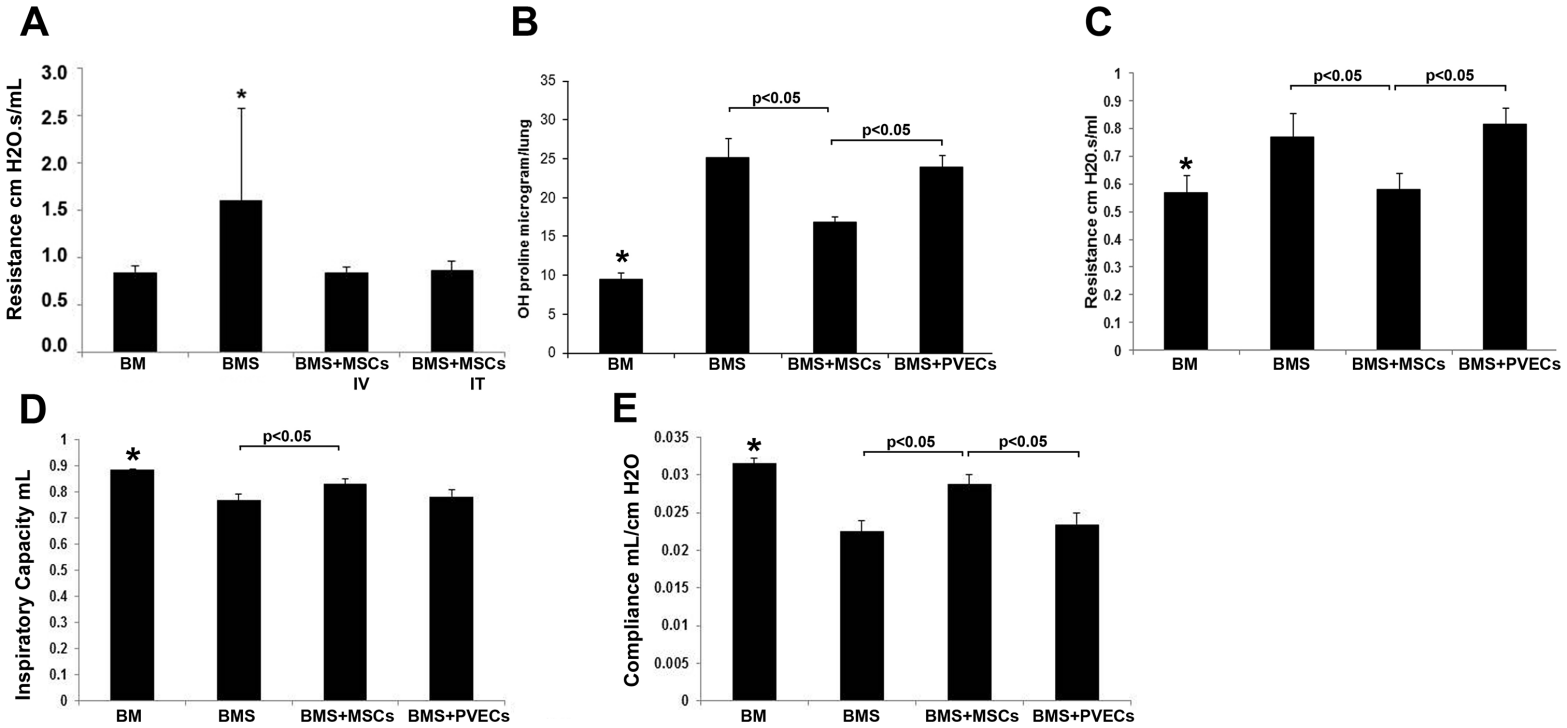
Early administration of MSC reduces post BMT manifestations of OB. Starting at 2 weeks post-BMT, MSCs or PVECs were administered IV or IT weekly and evaluated on day 90 post-BMT. **A.** Interim analysis at day 60 post-BMT shows equivalent efficacy of MSCs administered by either the IV or IT route as assessed by improved (reduced) airway resistance. N = 4–6/group, *p = 0.03 vs all other groups. **B.** Day 90 post-BMT hydroxyproline levels indicating degree of fibrosis is significantly reduced by administration of MSCs. *p<0.05 vs all other groups; other significant differences indicated on graph. N = 4–7/group, IV and IT combined) **C, D, E.** Improved day 90 post-BMT airway resistance, inspiratory capacity and compliance, respectively, by administration of MSCs. *p<0.05 vs BMS and BMS+PVECs; other significant differences indicated on graph. N = 4–7/group, IV and IT combined. PVECs = pulmonary vein endothelial cells used as control cells. All data are pooled from 2 experiments.

**Figure 3:**
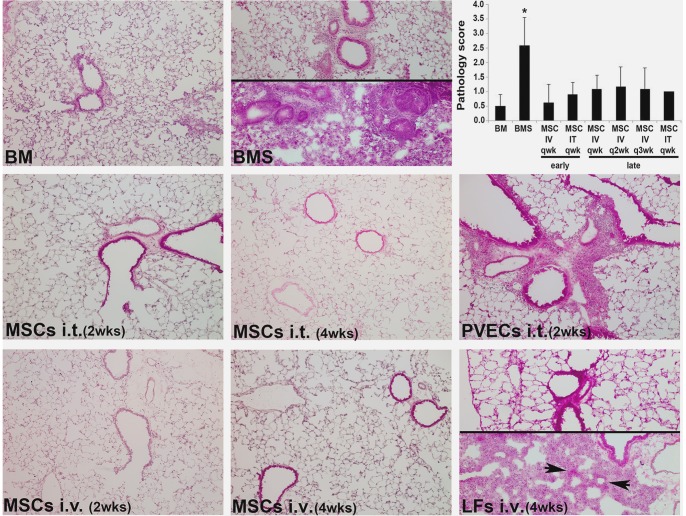
Administration of MSC post-BMT reduces OB histopathology. Representative H&E stained lung cryosections of day 90 post-BMT shown for mice receiving T cell-depleted bone marrow only (**BM**); BM and allogeneic splenocytes (**BMS**; OB group). The split panels show the range of injury in this model. **MSCs IV** or **IT** as indicated, beginning at 2 or 4 weeks and given weekly; **PVECs IT** beginning at 2 weeks and given weekly; **LFs IV** beginning at 4 weeks and given weekly until day 90. The split panels show the range of injury seen in this group similar to the BMS group. Original magnification 200× (20× objective lens). The top right corner panel shows semiquantitative pathology scores; n = 4–6/group pooled from 4 experiments.

Mice receiving BMS were given either MSCs or PVECs weekly starting at 2 weeks post-BMT (i.e. early). Interim analysis at day 60 post-BMT demonstrated that MSCs were equally as effective when administered via either the IV or IT route as shown by reduced airways resistance in **Figure 2A**. However, repeated anesthesia for the IT administration took a toll on survival and subsequent data were combined. At day 90 post-BMT, consistent with our documented published mouse model, OB (BMS) mice exhibited increased levels of OH-proline, increased airways resistance, decreased inspiratory capacity and compliance (**Figure 2B, C, D&E**). Mice receiving MSCs had reduced levels of lung OH-proline, reduced airways resistance, increased inspiratory capacity and compliance compared to mice receiving control cells (PVECs) or medium only (BMS mice). Histopathologic examination showed extensive peribronchiolar inflammation in groups receiving BMS or PVECs after BMT (**Figure 3**); inflammation was not seen in mice given MSCs. Semi-quantitative pathologic scores were consistent with the above biochemical and physiologic parameters (graph in **Figure 3**). No ectopic tissue formation was found. Therefore, early administration of MSCs at a point when lung function begins to decline had a significant beneficial impact and led to reduction of OB manifestations.

### Late administration of MSCs attenuates airway injury and improves PFT parameters after BMT

**Figure 4:**
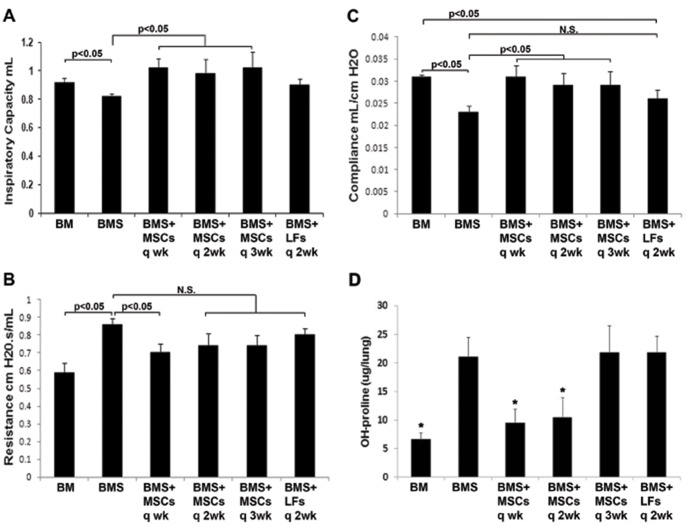
Late administration of MSC post-BMT reduces manifestations of OB. Starting at 4 weeks post-BMT, MSCs were administered IV every 1, 2 or 3 weeks until day 90. **A**. Inspiratory Capacity; **B**. Resistance; **C**. Compliance; D. OH-proline. Significant differences are indicated on graph. N = 6–14/group pooled from 2 experiments. LFs = mouse lung fibroblasts used as control cells.

Mice receiving BMS were given either MSCs or LFs IV starting at 4 weeks post-BMT (i.e. late). To determine how frequently cells needed to be administered for benefit to be achieved, cell therapy was compared for administration every 1, 2 or 3 weeks. Mice receiving MSCs exhibited improved inspiratory capacity and lung compliance compared to BMS mice and those receiving control cells, even if cells were given only every 3 weeks (**Figure 4A and C**). The increase in resistance and OH-proline was attenuated in mice given MSCs every week (**Figure 4B & D**); q2week and q3week MSC groups also had lower resistance but it was not statistically significant compared to BMS mice. Mice given late administration of MSCs had histologically normal lungs (**Figure 3**). Therefore, late administration of MSCs at a point when lung function begins to decline had a significant beneficial impact and led to reduction of OB manifestations.

### Increased frequencies of AAMs in lungs of mice receiving MSCs

**Figure 5:**
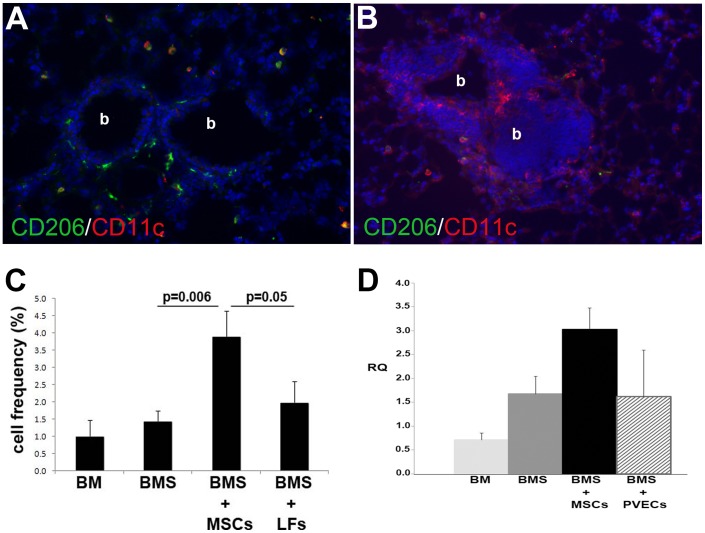
Increased expression of markers of AAMs in lungs of mice given MSC post-BMT. Starting at 4 weeks post-BMT, MSCs (**A**) or LFs (**B**) were administered IV weekly and evaluated on day 90 post-BMT. Lung cryosections were immunofluorescently stained for CD11c with CD206 (**A, B**) and frequency of CD206^+^ cells was determined as a percent of total nucleated cells (data in **C** shown as mean ± SE, n = 5–7/group pooled from 2 experiments). Images are at 400X total magnification; “b” indicates bronchiolar airway. In **D**, cell therapy (IT, weekly) was started at 2 weeks and lungs examined on day 90 post-BMT by qRT-PCR for CD206 (n = 3/group).

In order to determine the possible mechanism by which MSCs were ameliorating OB after BMT, lungs were examined for presence of AAMs. Immunofluorescence staining showed increased frequencies of CD206+ cells, indicative of AAMs, in the mice receiving MSCs (**Figure 5 A**) compared to those receiving control LFs (**Figure 5B**) or no cell therapy (compiled data shown in **Figure 5C**). **Figure 5D** shows that qRT-PCR analysis for CD206 expression in the lungs was also consistent with the increased presence of AAMs in MSC-treated mice. Flow cytometric analysis of lungs from a small cohort of mice also showed increased CD206^+^ macrophages as well as an increase in CD4^+^/FoxP3^+^ cells albeit not reaching statistical significance due to small sample size (**Figure S2**).

### Differential expression of cytokines identify TSG-6, FGF-7 and IL-1ra as potential mediators of anti-OB activity

In order to demonstrate that the beneficial effect of MSCs on OB was MSC-related, we compared the cytokine secretion profile of MSCs with PVECs and LFs that were used as control cells. **Table 1** shows the mediators that were detected among the many cytokines we were able to test for (listed in methods). As shown in Table 1, several mediators were produced by all 3 cell types, namely, IL-6, CXCL1, and PAI-1. PGE2 was produced by both MSCs and LFs at equivalent levels. IL-1ra was also found in MSCs and LFs but to a higher level in MSCs. The only mediators found to be exclusively secreted by the MSCs were TSG6, FGF7 (KGF) and GM-CSF. Although IL-10 secretion by the MSCs was not detected in vitro, it does not negate their ability to secrete it in vivo after infusion. Analysis of IL-10 expression in lungs of mice given MSCs weekly starting at 2 weeks post-BMT, as assessed by qRT-PCR, revealed no increase (in fact it was lower) compared to mice given control, or no, cell therapy **(Figure S3)**. Thus, potential effectors of the immunomodulatory activity of MSCs are TSG6, FGF7, GM-CSF and possibly IL-1ra.

**Table 1:** Comparison of cytokine secretion.

Cells	PGE2	IL-1RA	TSG6	FGF7	GM-CSF	IL-6	CXCL1	PAI-1	G-CSF	LIX
MSC	+++	++	++	+	+	+	+++	++++	−	−
PVEC	−	−	−	−	−	+	+++	++++	++	+
LF	+++	+	−	−	−	+	+++	+	+/−	−

Key:+++ = >1,000 pg/mL;++ = 100–1000 pg/mL;+ = 10–99 pg/mL; +/− = <10pg/mL;

− = not detectable.

## Discussion

In this study, we have shown that cell therapy with MSCs can ameliorate OB in a murine BMT model. To our knowledge, this is the first report to explore the impact of delayed administration of MSCs on development of OB in an experimental model system. Our intent was not to prove the mechanism of action but to demonstrate the potential of MSCs to treat OB and to determine some parameters of cell administration. In our study, we have shown the beneficial impact of MSCs not only when administered early but also when administered late, after the onset of OB has been confirmed by decline in PFTs.

In our current study, the MSCs used were syngeneic to the donor (i.e. autologous to the new immune system, but allogeneic to the recipient). In the clinical experience of MSCs for HSCT, the MSCs have been third party [26]. They are considered to be relatively immunoprivileged and their allogenicity has not been an issue in several studies [27], [28]. However, many studies have shown allogeneic MSCs to be immunogenic, especially when pretreated with IFNγ ([29] and reviewed in [30]). IFNγγ pretreatment increases expression of immunosuppressive molecules by MSCs but also induces MHC Class II expression, enhancing their rejection [30]. There are certainly pros and cons in using either auto- or allogeneic MSCs, with or without IFNγ induction, encompassing their engraftment, immunosuppressive abilities and unwanted accelerated organ rejection, especially in the presence of donor-specific alloantibodies. The choice of optimal MSC preparation may depend on whether the disease indication is acute or chronic. It is also not resolved whether it is desirable to have MSCs persist long term at the site of injury.

MSCs in our study were administered in small doses at regular intervals to achieve the desired effect. This allows the possibility to achieve a favorable response by using smaller numbers of cells in a patient population using a strategy that can be very helpful in the clinical setting [10], [31] where MSCs are more easily administered via an intravenous versus an intra-tracheal route. IV administration was used in recently reported early phase clinical trials for idiopathic pulmonary fibrosis and COPD and was shown to be safe [32], [33]. IT administration was also safe [34]. We compared the IT to the IV route of administration and found in interim analysis that there was no difference when administration began early (i.e. at 2 weeks). However, we found that the mice did not tolerate the multiple anesthesias well in the context of the IT procedure, regardless of cell type given, at the late time points when lung function had declined significantly. Therefore, the IV route was considered the best option for late administration.

The administration of exogenous MSCs resulted in improved lung histology and pulmonary function tests. Administration of MSCs also resulted in increases of cells with morphology consistent with alternatively activated macrophages (AAMs) which have immunosuppressive properties [35]. In addition, MSC-treated mice had a statistical trend toward increased CD4+Foxp3+ cells, consistent with Tregs, as has been described earlier [36], including in a tracheal transplant model [37]. Generation of AAMs under the influence of MSCs has been reported [38], [39]. It is interesting that CD11c expression was low in the lungs of MSC-treated mice. CD11c is expressed by lung macrophages, in contrast to macrophages from other organs and tissues. To our knowledge, it has not been described that AAM (M2) macrophages in the lung lose CD11c expression. However, it is known that the lung environment affects CD11c expression/upregulation and that GM-CSF is one of the factors that stimulates CD11c expression by lung macrophages (or of macrophages from other areas that are exposed to the lung environment) [40]. Indeed, our cytokine analysis did show that the MSCs produced GM-CSF in culture. We hypothesize that MSC-derived GM-CSF has affected the phenotype of the recruited macrophages.

A recent study has shown the beneficial effect of MSCs in the orthotopic tracheal transplant OB model being due to MSC-derived PGE2 leading to increased IL-10 levels in the tracheal graft [41]. Increased IL-10 was also found in MSC-treated mice with a heterotopic tracheal transplant [42]. However, our cytokine analysis indicated that PGE2-mediated IL-10 increase was not likely the mechanism responsible since LFs also produced PGE2 but did not ameliorate OB. Furthermore, we found no increase in IL-10 in the recipient lungs of MSC-treated mice compared to controls. Future studies using neutralizing antibodies to PGE2 and/or IL-10 (or IL-10^−/−^ MSCs) are required to eliminate these mediators as effectors of donor MSCs in this model. Despite this, the in vitro production of PGE2 by our MSCs classifies them as anti-inflammatory MSC2 cells [43].

The beneficial effect of MSCs could be due to the immunoregulatory properties of the MSCs directly by virtue of the mediators they produce, or indirectly by the generation of Tregs and AAMs. Other possible mechanisms include mitochondrial transfer to rescue stressed cells and improve cellular bioenergetics and function to allow for more efficient repair, as has been elegantly demonstrated previously [44]. Oxidative stress of lung epithelium has been demonstrated in humans with OB after lung transplant and in mice with induced alloimmune activation [45], [46], [47]. In addition, we have previously shown that the lungs are under oxidative stress after BMT in our mouse model and that this could be normalized by treatment with KGF, a factor typically secreted by MSCs [48]. In our current study, we never found engraftment of MSCs in the lungs consistent with the findings of many other groups in acute lung injury models. We did not find any effect on donor BM engraftment as demonstrated by others (albeit in a non-myeloablative setting) [49], as all recipient mice were found to be >95% donor engrafted (data not shown). Perhaps this is due to the administration of cells at a time sufficiently removed from the peri-BMT period in our study.

Our cytokine analysis comparing MSCs to the control cells identified TSG6, FGF7, GM-CSF and possibly IL-1ra as potential mediators of the MSC effect, although neutralizing antibodies or knockout approaches would be required to confirm this. The Prockop group has identified TSG6 as an anti-inflammatory mediator produced by MSCs that can ameliorate LPS- and bleomycin-induced lung injury in mice when given systemically in the early stages of inflammation [50], [51]. MSCs injected into skin wounds controlled macrophage activation and limited fibrosis through a TSG6 mechanism in a murine model [52]. FGF7, also known as KGF, has been shown to be effective in preclinical models of lung injury including acute lung injury [53] and post-BMT-induced idiopathic pneumonia syndrome when given as a pretreatment [54]. In a recent study, human MSCs improved alveolar fluid clearance in human donor lungs (rejected for transplant), most likely by a KGF-mediated mechanism since anti-KGF neutralizing antibody abrogated the effect [12]. This demonstrated benefit could lead to an increase in the number of donor lungs suitable for transplantation. However, it has been demonstrated many times that KGF has little beneficial effect when given after injury, making its usefulness for OB less likely, although it is possible that it aids in preventing further injury, thus enabling repair. A recent study of repeated bleomycin-induced lung injury did demonstrate that a slight (3-day) delay in administering MSCs could ameliorate inflammation and fibrosis, possibly through secretion of IL-1RA [55]. IL-1ra has also been shown to mediate beneficial effects of MSCs in murine lung injury models, but, again, only when given as a pre-treatment [56]. Notwithstanding the non-exhaustive list of analytes we were able to assay for, TSG6 stands out as the most likely candidate for the MSC effect, although adjunct activity of the aforementioned cytokines cannot be ruled out.

Further studies will be needed to determine the impact of MSC administration in combination with immunosuppressive medications to further evaluate their potential application in lung transplant recipients. More studies on the minimal effective dose and the need for cells versus cell-derived factors are also needed. MSCs have also been shown to have antimicrobial properties against a variety of pathogens [57]. As lung transplant and HCT recipients are at increased risk of opportunistic infections due to immunosuppressive medications, this may provide an additional advantage. All these characteristics make MSCs an attractive management option for lung transplant recipients which merits further evaluation and will hopefully show some promising results in an ongoing clinical trial led by the Chambers group in Australia (trial #NCT01175655).

## Supporting Information

Figure S1 **Phenotypic analysis of BM-derived MSCs. MSCs were characterized by flow cytometry for the indicated surface markers.**
(TIF)

Figure S2 **Increased frequencies of CD4^+^/FoxP3^+^ (Treg) cells and CD206 macrophages (AAM) in lungs of MSC-treated mice.** MSCs or LFs were given weekly starting at 2 weeks post-BMT. Cells from collagenase-digested lungs were analyzed by flow cytometry for CD4 with FoxP3, and for CD206 with F/480. N = 3 pooled samples per group from 2 experiments.
(TIF)

Figure S3 **Increased IL-10 expression in lungs of all BMS mice regardless of cell therapy.** MSCs or PVECs were started at 2 weeks post-BMT. qRT-PCR was done on lungs at day 90 post-BMT. N = 3/group from 1 experiment. *P<0.03 vs all other groups.
(TIF)
