# Supplementary figures and images for: MSC Therapy Attenuates Obliterative Bronchiolitis after Murine Bone Marrow Transplant
Source: PLoS One. 2014 Oct 1;9(10):e109034. doi: 10.1371/journal.pone.0109034 (PMC4182803; doi:10.1371/journal.pone.0109034)

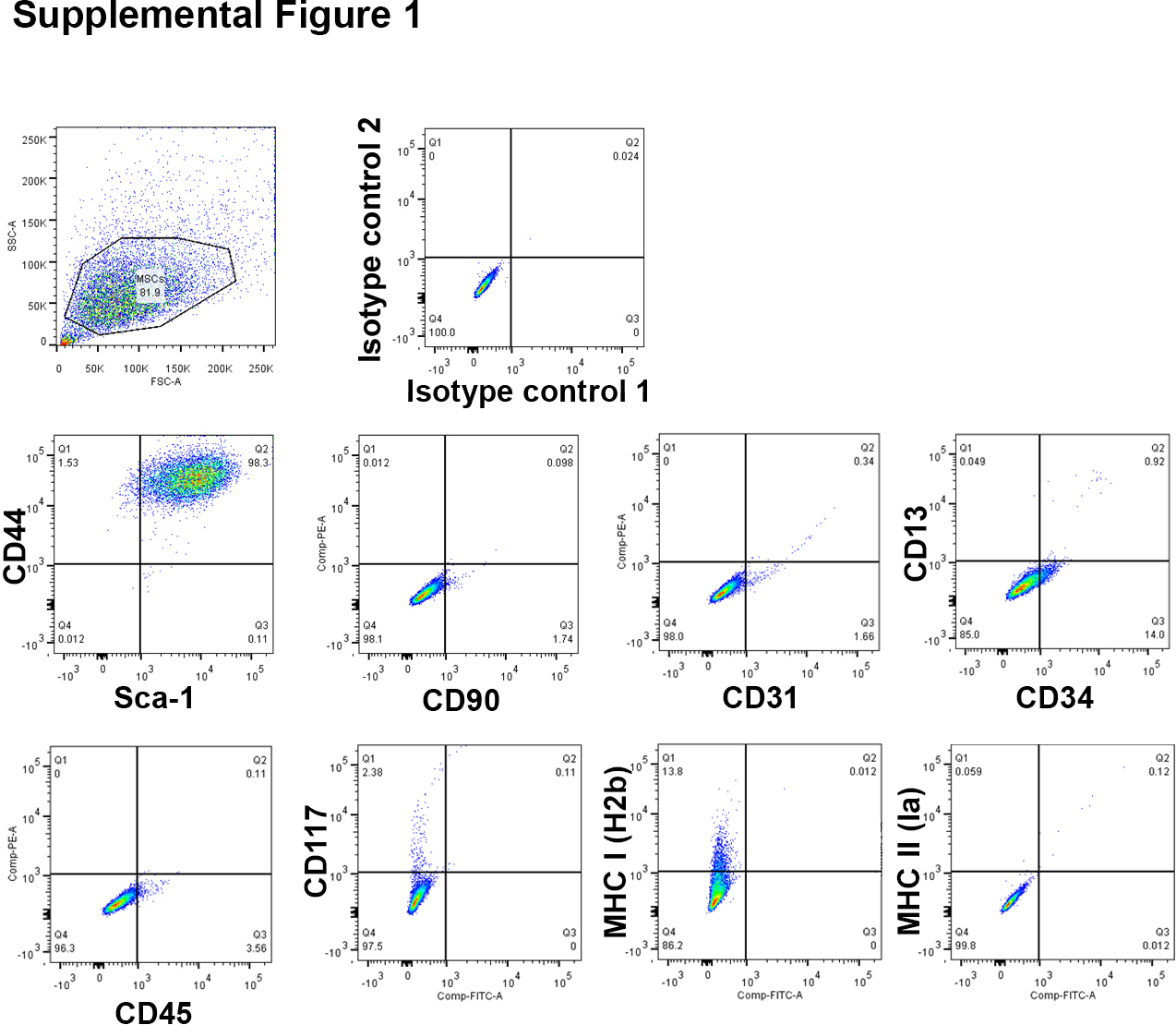

Supplement: Figure S1 — Phenotypic analysis of BM-derived MSCs. MSCs were characterized by flow cytometry for the indicated surface markers. (TIF) [file pone.0109034.s001.tif]

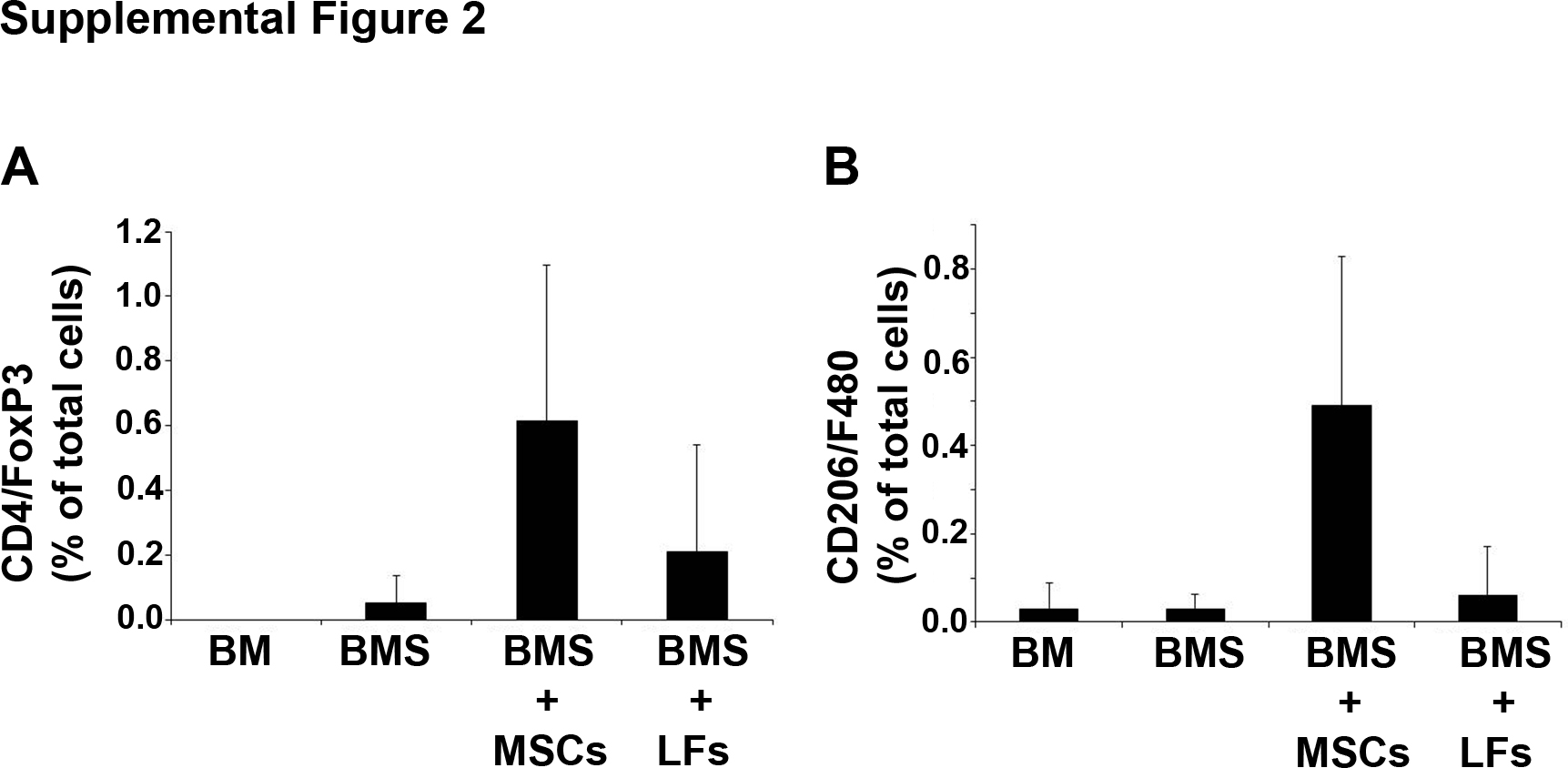

Supplement: Figure S2 — Increased frequencies of CD4+/FoxP3+ (Treg) cells and CD206 macrophages (AAM) in lungs of MSC-treated mice. MSCs or LFs were given weekly starting at 2 weeks post-BMT. Cells from collagenase-digested lungs were analyzed by flow cytometry for CD4 with FoxP3, and for CD206 with F/480. N = 3 pooled samples per group from 2 experiments. (TIF) [file pone.0109034.s002.tif]

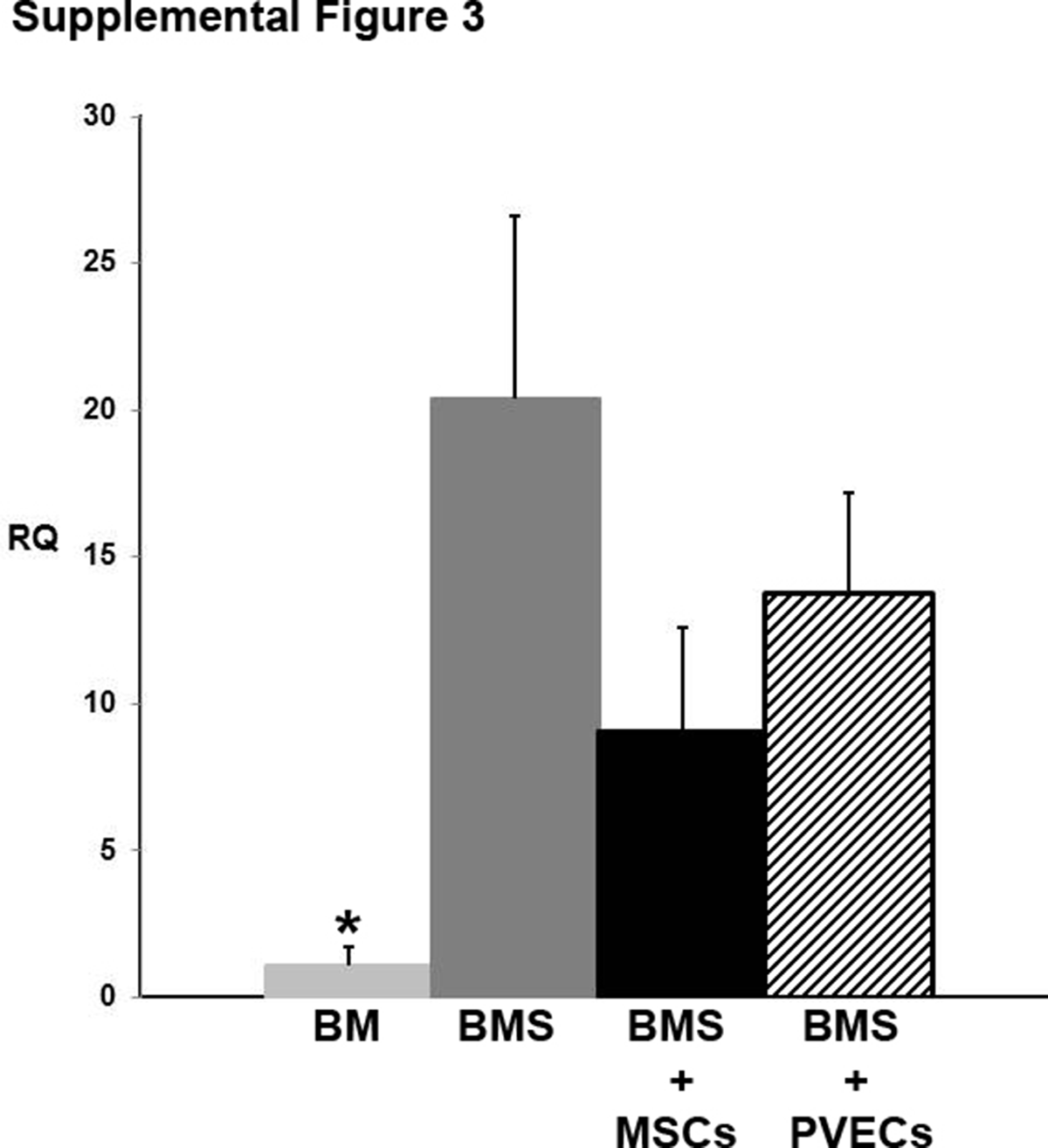

Supplement: Figure S3 — Increased IL-10 expression in lungs of all BMS mice regardless of cell therapy. MSCs or PVECs were started at 2 weeks post-BMT. qRT-PCR was done on lungs at day 90 post-BMT. N = 3/group from 1 experiment. *P<0.03 vs all other groups. (TIF) [file pone.0109034.s003.tif]
